# Impact of alkaline phosphatase on clinical outcomes in patients with ischemic stroke: a nationwide registry analysis

**DOI:** 10.3389/fneur.2024.1336069

**Published:** 2024-02-14

**Authors:** Zhaobin Wang, Jing Li, Jing Jing, Zhe Zhang, Qin Xu, Tao Liu, Jinxi Lin, Yong Jiang, Yongjun Wang, Anxin Wang, Xia Meng

**Affiliations:** ^1^Affiliated Hospital of Hebei University, Baoding, China; ^2^Clinical Medical College, Hebei University, Baoding, China; ^3^Department of Neurology, Beijing Tiantan Hospital, Capital Medical University, Beijing, China; ^4^Puyang Oilfield General Hospital, Puyang, China; ^5^China National Clinical Research Center for Neurological Diseases, Beijing Tiantan Hospital, Capital Medical University, Beijing, China; ^6^Beijing Advanced Innovation Center for Biomedical Engineering, School of Biological Science and Medical Engineering, Beihang University, Beijing, China

**Keywords:** alkaline phosphatase, mortality, disability, poor functional outcomes, stroke

## Abstract

**Background:**

Data on the association between serum alkaline phosphatase (ALP) levels and clinical outcomes in patients with ischemic stroke (IS) are inconsistent and limited. Therefore, this study aimed to investigate the correlation between ALP and prognosis in patients with IS.

**Methods:**

Patients with acute ischemic stroke (AIS) or transient ischemic attack (TIA) from the Third China National Stroke Registry were divided into four groups according to the quartiles of serum ALP levels on admission. Cox proportional hazards and logistic regression models were used to evaluate the correlation between ALP and the risk of all-cause mortality, disability (modified Rankin Scale (mRS) score 3–5), and poor functional outcomes (mRS score 3–6).

**Results:**

A total of 11,405 patients were included in the study. Higher levels of ALP were associated with all-cause mortality at 3 months (adjusted hazard ratio [HR] per standard deviation [SD]: 1.16; 95% confidence interval (CI): 1.07–1.27; *p* = 0.001) and 1 year (adjusted HR: 1.11; 95% CI: 1.03–1.20; *p* = 0.010). At the 3-month follow-up, each SD increase of ALP was associated with a 12 and 14% higher risk of disability (adjusted odds ratio (OR): 1.12; 95% CI: 1.06–1.18; *p* < 0.001) and poor functional outcomes (adjusted OR: 1.14; 95% CI: 1.08–1.20; p < 0.001). Similar results were observed at the 1-year follow-up. Higher ALP levels were associated with an increased risk of all-cause mortality, disability, and poor functional outcomes in patients with “others” subtypes (including other determined etiology and undetermined etiology) (*p* < 0.05).

**Conclusion:**

Elevated ALP levels were associated with an increased risk of all-cause mortality, disability, and poor function outcomes in patients with IS. Heterogeneity was observed among the subtypes of different etiologies.

## 1 Introduction

Stroke is the second leading cause of death and the third leading cause of disability worldwide (1, 2). Globally, China faces the most significant stroke burden, with ischemic stroke (IS) accounting for over 82% of all stroke cases (3). Therefore, identifying reliable blood markers for stroke prognosis is crucial for optimizing healthcare resource allocation (4).

Alkaline phosphatase (ALP), a widely expressed enzyme in human tissues, has been implicated in vascular calcification and the development of atherosclerosis (5–7). Inhibition of ALP has been shown to prevent the formation of vascular atherosclerosis (8). Traditionally recognized as a marker for skeletal or hepatobiliary dysfunction (9, 10), ALP is now considered indicative of atherosclerosis and inflammatory responses (6, 11). Studies have indicated that elevated serum ALP levels are linked to increased atherosclerosis in coronary and peripheral arteries and that higher ALP levels are independently associated with the risk of cardiovascular disease (CVD) and mortality events (12–14). However, conflicting findings exist among epidemiological investigations regarding the association between higher serum ALP levels and adverse clinical outcomes in stroke patients. Multiple studies have suggested that high serum ALP levels are associated with an increased incidence of stroke, higher post-stroke mortality rates, and poor functional outcomes (15–19). However, other studies have concluded that there was no significant association between increased ALP levels and poor functional outcomes (18, 20). Therefore, at present, there is no research consensus on the association between ALP levels and clinical outcomes in patients with stroke, and studies on this topic have been limited. Furthermore, the different etiologies of stroke have not yet been examined.

Therefore, this study aims to utilize a large sample from the China National Stroke Registry III (CNSR-III) to investigate the correlation between serum ALP levels and clinical outcomes (mortality, disability, and poor functional outcomes) in patients with acute ischemic stroke (AIS) and transient ischemic attack (TIA), analyze the association between serum ALP levels and stroke subtypes, and further explore the underlying mechanism of ALP.

## 2 Methods

### 2.1 Study population

CNSR-III is a nationwide prospective registry of consecutive patients with AIS or TIA. Patients were enrolled from 201 hospitals between August 2015 and March 2018. The detailed design and description of the CNSR-III have been published previously (21). The inclusion criteria were as follows: (1) age 18 years or older and (2) diagnosis of ischemic stroke or TIA within 7 days from the onset of symptoms to enrollment. The exclusion criteria were as follows: (1) silent cerebral infarction with no manifestation of symptoms and signs, and (2) refusal to participate in the registry.

The CNSR-III study was approved by the ethics committee of Beijing Tiantan Hospital (NO. KY2015-001-01), and written informed consent was obtained from patients or their legally authorized representatives. The study complied with the principles of the Declaration of Helsinki.

### 2.2 Data collection and management

After admission, the participants were collected by a trained neurologist in the hospital and the following baseline data were recorded: age, sex, body mass index (BMI, calculated as kg/m^2^), smoking and alcohol consumption status, medical history (previous diabetes, hypertension, dyslipidemia, coronary heart disease, and stroke), treatment during hospitalization (intravenous thrombolysis, mechanical thrombectomy, antiplatelet aggregation therapy, anticoagulation therapy, and lipid-lowering therapy), National Institutes of Health Stroke Scale (NIHSS) score on admission, and pre-stroke modified Rankin Scale (mRS) score. In addition, serum ALP, alanine aminotransferase (ALT), and aspartate aminotransferase (AST) were obtained through venous puncture within 24 h. Total cholesterol (TC), high-density lipoprotein (HDL), low-density lipoprotein (LDL), triglyceride (TG), estimated glomerular filtration rate (eGFR), and high-sensitivity C-reactive protein (hs-CRP) samples were transported to the central laboratory of Beijing Tiantan Hospital for centralized testing through the cold chain.

All imaging data were collected on disks in the DICOM format, analyzed by two professional neurologists, and classified by etiological TOAST (Org10172 trial in the treatment of acute stroke). As there were a few patients with a stroke of other determined etiology, these patients were combined as a stroke of undetermined etiology and defined together as the “others.” Hence, patients in this study were classified into four subtypes: large-artery atherosclerosis (LAA), cardioembolism, small-vessel occlusion, and others (including other determined etiology and undetermined etiology).

### 2.3 Patient follow-up and clinical outcome assessment

The clinical outcomes were obtained by trained research coordinators who were unaware of the participants’ baseline characteristics, through a face-to-face interview at 3 months and via the telephone at 1 year after the onset of symptoms. Clinical outcomes included all-cause mortality, disability, and poor functional outcomes at the 3-month and 1-year follow-up. All-cause mortality was either confirmed by a death certification from the attended hospital or the local citizen registry, and the mRS score ranged from 0 (no symptoms) to 6 (death); poor functional outcome was determined by an mRS score of 3–6, while major disability was determined by an mRS score of 3–5.

### 2.4 Statistical analysis

This study’s population characteristics were presented as medians (interquartile ranges, IQRs) or numbers (proportions) by quartiles of serum ALP levels. The associations of ALP with all-cause mortality, disability, and poor functional outcomes at 3 months and 1 year were assessed. For all-cause mortality, we used the Kaplan–Meier method to estimate the cumulative incidence in the ALP quartile groups, and the difference across groups was compared using the log-rank test. Hazard ratios (HRs) and 95% confidence intervals (CIs) were estimated using the Cox proportional hazards models. The proportional hazards assumption was checked using Schoenfeld residuals over time, and no deviations from the assumption were found. For disability and poor functional outcomes, odds ratios (ORs) with 95% CIs were estimated using logistic regression models. Serum ALP was included in the models, both as a categorized variable (in quartiles) and as a continuous variable.

Based on the clinical experience and relevant literature (16–18), we selected covariates and fitted three adjusted models. Model 1 was adjusted for age and gender. Model 2 was further adjusted for BMI, current smoking, heavy drinking, pre-stroke mRS score, TOAST classification, hypertension, diabetes, dyslipidemia, coronary heart disease, and previous stroke. Model 3 was further adjusted for antiplatelet agents, anticoagulant agents, estimated glomerular filtration rate, and high-sensitivity C-reactive protein. To visualize the potential non-linear associations of serum ALP with death, disability, and poor functional outcomes, we constructed restricted cubic splines with three knots at the 10th, 50th, and 90th percentiles. Stratified analyzes were performed in the subgroups of TOAST types. All statistical analyzes were conducted using SAS version 9.4 (SAS Institute Inc., Cary, NC, United States) and R software version 4.1.3 (R Foundation for Statistical Computing). The statistical significance was determined as two-sided *p-*values of <0.05.

## 3 Results

### 3.1 Baseline characteristics

We excluded 3,761 patients from the initial 15,166 patients due to underlying conditions, such as liver disease (*n* = 100), kidney disease (*n* = 131), arthritis (*n* = 329), cancer (*n* = 134), infection within 2 weeks before admission (*n* = 450), or missing ALP values (*n* = 2,337), as well as mRS scores at the 1-year (*n* = 349) or 3-month (*n* = 170) follow-up (Supplementary Figure S1). The final analysis encompassed 11,405 patients. The baseline characteristics of both included and excluded patients are presented in Supplementary Table S1, demonstrating a balanced distribution between the two groups. Table 1 summarizes the baseline characteristics of the included patients, with a median age of 63 (54.0–70.0) years. Among the included patients, 10,525 (92.3%) were diagnosed with AIS, 7,784 (68.3%) were male patients, 3,605 (31.6%) were current smokers, 1,628 (14.3%) were heavy drinkers, 949 (8.3%) received intravenous thrombolysis, and 32 (0.3%) of them underwent mechanical thrombectomy. The median NIHSS score was 3 (1.0, 6.0). In the higher quartile ALP groups, patients were more likely to have a history of hypertension, coronary heart disease, and stroke, while they were less likely to have diabetes, be smokers, and consume alcohol. In TOAST classification, all ALP quartile groups had a relatively higher number of LAA and undetermined etiology stroke patients. The levels of hs-CRP increased with the increase in serum ALP levels (Table 1).

**Table 1:** Baseline characteristics by alkaline phosphatase quartile.

Characteristics	Quartiles of ALP					*p*-value
	Overall	Q1 (<62)	Q2 (62–75)	Q3 (75–91)	Q4 (≥91)	
*n*	11,405	2,794	2,864	2,894	2,853	
AIS	10,525 (92.3)	2,531 (90.6)	2,637 (92.1)	2,687 (92.8)	2,670 (93.6)	<0.001
Age, years	63.0 [54.0, 70.0]	62.0 [54.0, 71.0]	62.0 [54.0, 69.0]	62.0 [54.0, 70.0]	63.0 [55.0, 70.0]	0.111
Men, *n* (%)	7,784 (68.3)	2080 (74.4)	2065 (72.1)	1991 (68.8)	1,648 (57.8)	<0.001
Body mass index, kg/m^2^	24.5 [22.6, 26.5]	24.5 [22.6, 26.4]	24.5 [22.6, 26.4]	24.5 [22.6, 26.6]	24.5 [22.5, 26.6]	0.920
Current smoking, *n* (%)	3,605 (31.6)	863 (30.9)	926 (32.3)	970 (33.5)	846 (29.7)	0.010
Heavy drinking, *n* (%)	1,628 (14.3)	487 (17.4)	426 (14.9)	417 (14.4)	298 (10.4)	<0.001
Prestroke mRS score	0.0 [0.0, 1.0]	0.0 [0.0, 0.0]	0.0 [0.0, 1.0]	0.0 [0.0, 1.0]	0.0 [0.0, 1.0]	0.008
NIHSS score at admission	3.0 [1.0, 6.0]	3.0 [1.0, 5.0]	3.0 [1.0, 5.0]	3.0 [1.0, 6.0]	4.0 [1.0, 6.0]	<0.001
TOAST classification, *n* (%)						0.605
Large-artery atherosclerosis	2,898 (25.4)	699 (25.0)	701 (24.5)	737 (25.5)	761 (26.7)	
Cardioembolism	691 (6.1)	188 (6.7)	170 (5.9)	168 (5.8)	165 (5.8)	
Small-vessel occlusion	2,392 (21.0)	579 (20.7)	604 (21.1)	628 (21.7)	581 (20.4)	
Other determined etiology	147 (1.3)	38 (1.4)	42 (1.5)	38 (1.3)	29 (1.0)	
Undetermined etiology	5,277 (46.3)	1,290 (46.2)	1,347 (47.0)	1,323 (45.7)	1,317 (46.2)	
Medical history, *n* (%)						
Hypertension	7,123 (62.5)	1,695 (60.7)	1778 (62.1)	1810 (62.5)	1840 (64.5)	0.029
Diabetes mellitus	2,662 (23.3)	661 (23.7)	659 (23.0)	687 (23.7)	655 (23.0)	0.844
Dyslipidemia	861 (7.5)	233 (8.3)	184 (6.4)	230 (7.9)	214 (7.5)	0.039
Previous stroke	2,527 (22.2)	592 (21.2)	643 (22.5)	636 (22.0)	656 (23.0)	0.411
Coronary heart disease	1,189 (10.4)	283 (10.1)	281 (9.8)	290 (10.0)	335 (11.7)	0.065
Treatment in hospital, *n* (%)						
Intravenous thrombolysis	949 (8.3)	296 (10.6)	229 (8.0)	231 (8.0)	193 (6.8)	<0.001
Mechanical thrombectomy	32 (0.3)	12 (0.4)	9 (0.3)	5 (0.2)	6 (0.2)	0.258
Antiplatelet agents	11,002 (96.5)	2,682 (96.0)	2,743 (95.8)	2,804 (96.9)	2,773 (97.2)	0.010
Anticoagulant agents	1,191 (10.4)	307 (11.0)	275 (9.6)	289 (10.0)	320 (11.2)	0.138
Lipid-lowering agents	10,889 (95.5)	2,663 (95.3)	2,726 (95.2)	2,760 (95.4)	2,740 (96.0)	0.434
Laboratory tests						
TC, mmol/L	4.2 [3.4, 4.9]	4.1 [3.4, 4.8]	4.1 [3.4, 4.8]	4.2 [3.4, 5.0]	4.2 [3.5, 5.0]	<0.001
HDL-C, mmol/L	1.1 [0.9, 1.3]	1.1 [0.9, 1.3]	1.1 [0.9, 1.3]	1.1 [0.9, 1.3]	1.1 [0.9, 1.3]	0.006
LDL-C, mmol/L	2.5 [1.9, 3.1]	2.4 [1.8, 3.1]	2.4 [1.9, 3.1]	2.5 [1.9, 3.1]	2.5 [1.9, 3.2]	0.004
TG, mmol/L	1.4 [1.0, 1.9]	1.3 [1.0, 1.8]	1.4 [1.0, 1.9]	1.4 [1.0, 2.0]	1.4 [1.1, 2.0]	<0.001
ALT, U/L	18.0 [13.0, 25.0]	17.0 [12.0, 23.0]	17.7 [13.0, 25.0]	18.0 [13.0, 25.0]	19.0 [14.0, 28.0]	<0.001
AST, U/L	19.0 [16.0, 24.0]	18.0 [15.0, 22.6]	19.0 [15.3, 23.9]	19.0 [16.0, 24.0]	20.0 [16.0, 26.0]	<0.001
eGFR, mL/min/1.73 m^2^	93.2 [82.0, 101.8]	93.0 [81.4, 102.0]	93.5 [82.8, 102.0]	93.5 [82.5, 101.7]	93.0 [81.4, 101.8]	0.465
hs-CRP, mg/L	1.8 [0.8, 4.6]	1.3 [0.7, 3.5]	1.6 [0.8, 4.2]	1.9 [0.9, 4.7]	2.3 [1.0, 5.7]	<0.001

Continuous variables are expressed as median (interquartile range). Categorical variables are expressed as frequency (%). AIS, acute ischemic stroke; ALP, alkaline phosphatase; ALT, alanine aminotransferase; AST, aspartate aminotransferase; eGFR, estimated glomerular filtration rate; HDL-C, high-density lipoprotein cholesterol; hs-CRP, high-sensitivity C-reactive protein; LDL, low-density lipoprotein cholesterol; mRS, modified Rankin Scale; NIHSS, National Institutes of Health Stroke Scale; TC, total cholesterol; TG, triglycerides; TOAST, Trial of ORG 10172 in Acute Stroke Treatment.

### 3.2 All-cause mortality

**Figure 1:**
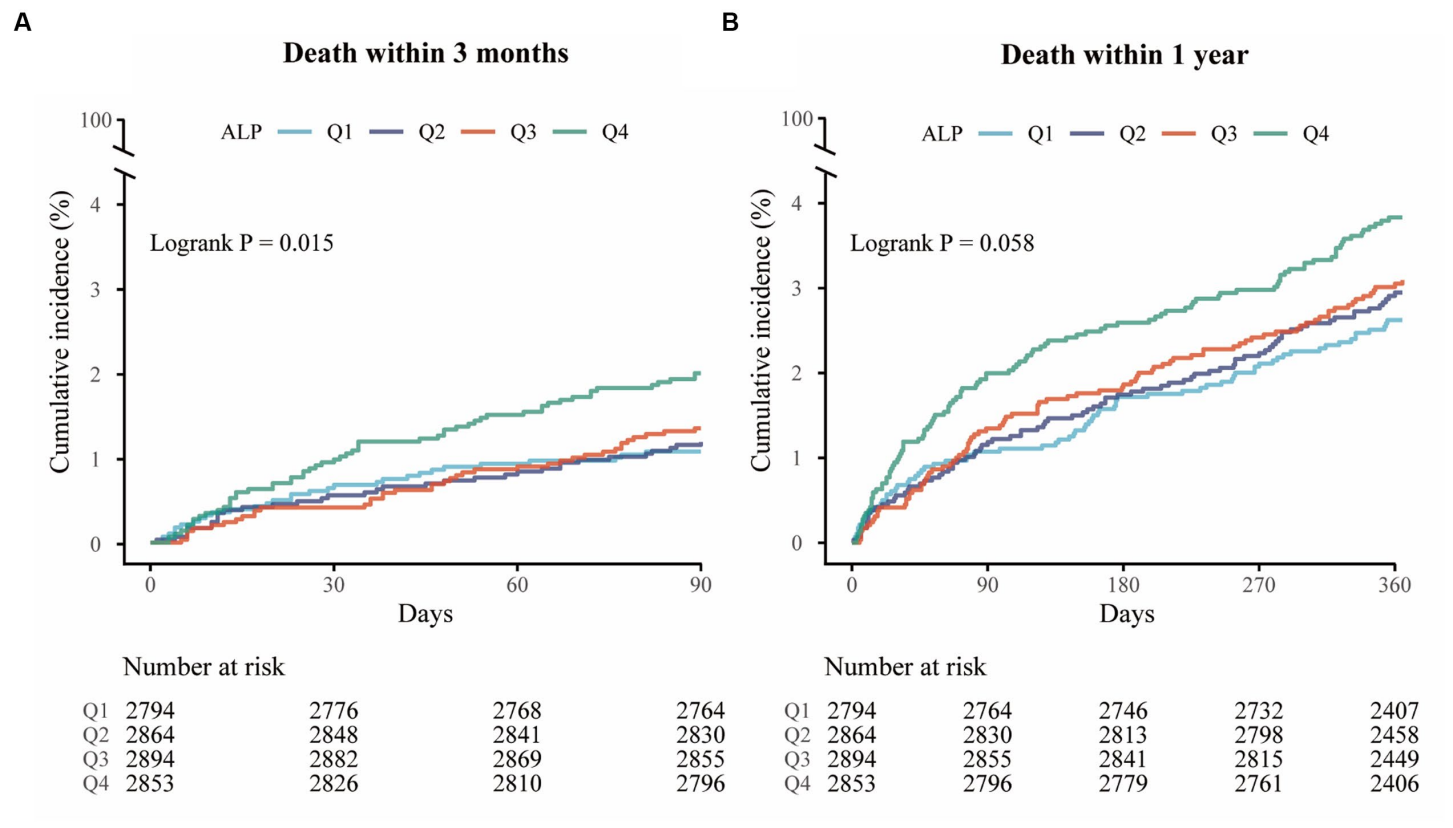
Kaplan–Meier curves for all-cause mortality. **(A)** Kaplan–Meier curves for all-cause mortality within 3 months (log-rank test; *p* = 0.015). **(B)** Kaplan–Meier curves for all-cause mortality within 1 year (log-rank test; *p* = 0.058).

**Figure 2:**
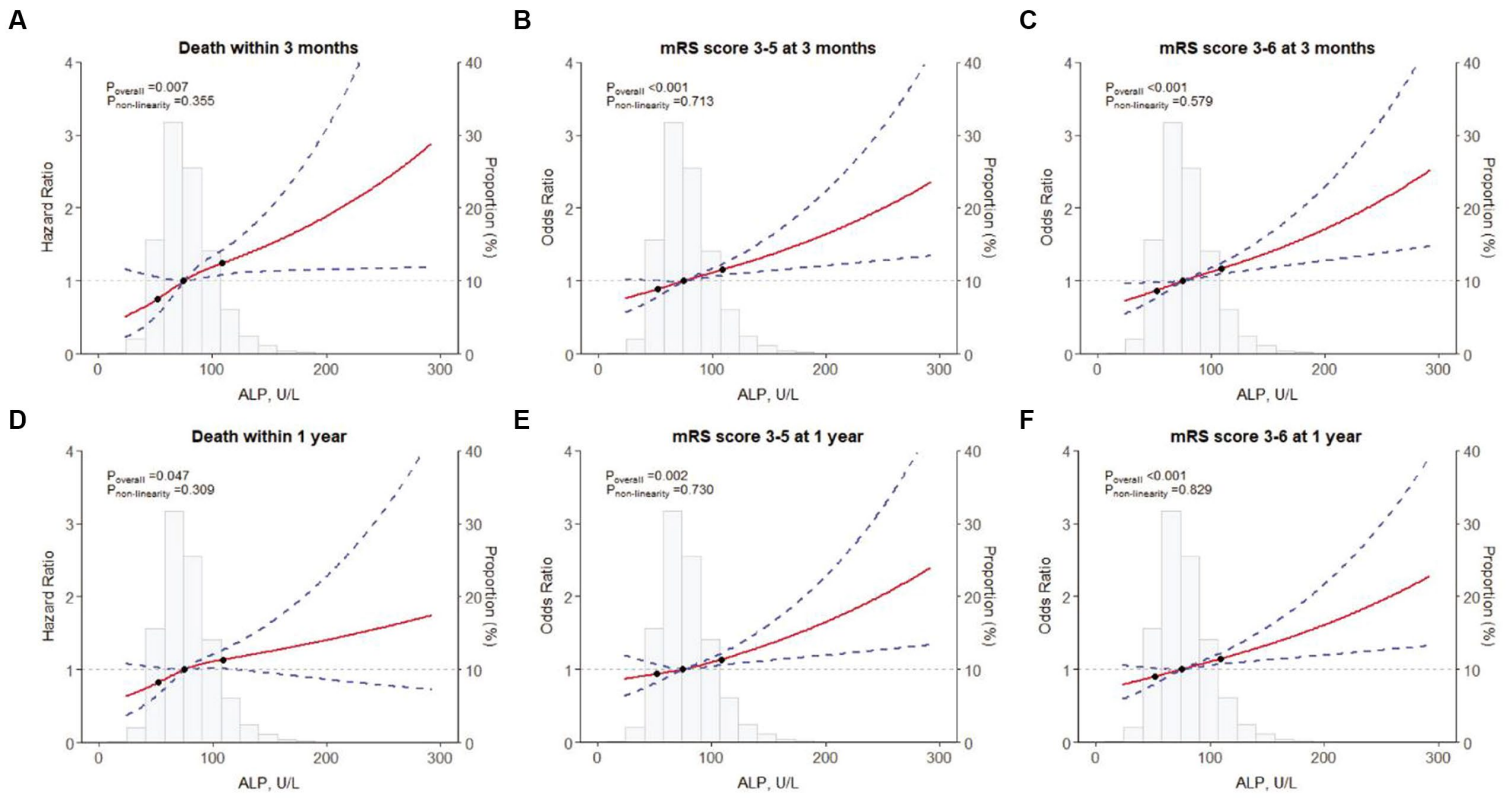
Restricted cubic spline for associations between ALP and clinical outcomes. **(A)** Death within 3 months; **(B)** an mRS score of 3–5 at 3 months; **(C)** an mRS score of 3–6 at 3 months; **(D)** Death within 1 year; **(E)** an mRS score of 3–5 at 1 year; **(F)** an mRS score of 3–6 at 1 year. ALP, alkaline phosphatase; mRS, modified Rankin Scale.

A total of 160 (1.4%) and 355 (3.1%) patients died during the 3-month and 1-year follow-up, respectively. The Kaplan–Meier curves showed that the cumulative incidence of all-cause mortality increased in patients with higher serum ALP levels within 3-month (log-rank *p* = 0.015) and 1-year (log-rank *p* = 0.058) follow-up (Figure 1). Higher levels of ALP were associated with all-cause mortality at 3 months (adjusted HR per standard deviation [SD]: 1.16; 95% CI: 1.07–1.27; *p* = 0.001) and 1 year (adjusted HR: 1.11; 95% CI: 1.03–1.20; *p* = 0.010) (Table 2). In addition, there was a linear correlation between the increase in ALP and all-cause mortality (*p* < 0.001; Figures 2A,D).

**Table 2:** Associations of alkaline phosphatase with all-cause mortality, disability, and poor functional outcomes.

	Events, *n* (%)	Unadjusted	*p*	Model 1	*p*	Model 2	*p*	Model 3	*p*
*At 3 months*									
Death									
Per SD increase	160 (1.4)	1.19 (1.10, 1.28)	<0.001	1.16 (1.07, 1.26)	<0.001	1.16 (1.07, 1.25)	<0.001	1.16 (1.07, 1.27)	0.001
Q1	30 (1.1)	Reference		Reference		Reference		Reference	
Q2	34 (1.2)	1.11 (0.68, 1.81)	0.690	1.16 (0.71, 1.89)	0.564	1.10 (0.67, 1.80)	0.697	1.10 (0.67, 1.80)	0.699
Q3	39 (1.4)	1.25 (0.78, 2.02)	0.350	1.27 (0.79, 2.04)	0.334	1.23 (0.76, 1.98)	0.403	1.23 (0.77, 1.99)	0.388
Q4	57 (2.0)	1.87 (1.20, 2.91)	0.006	1.83 (1.17, 2.87)	0.008	1.73 (1.11, 2.72)	0.016	1.63 (1.03, 2.56)	0.036
mRS score 3–5									
Per SD increase	1,354 (12.0)	1.15 (1.10, 1.21)	<0.001	1.13 (1.07, 1.19)	<0.001	1.13 (1.07, 1.19)	<0.001	1.12 (1.06, 1.18)	<0.001
Q1	290 (10.5)	Reference		Reference		Reference		Reference	
Q2	316 (11.2)	1.07 (0.91, 1.27)	0.418	1.08 (0.91, 1.28)	0.374	1.06 (0.89, 1.26)	0.504	1.06 (0.89, 1.26)	0.495
Q3	351 (12.3)	1.20 (1.01, 1.41)	0.034	1.19 (1.00, 1.40)	0.046	1.16 (0.98, 1.37)	0.086	1.17 (0.99, 1.39)	0.067
Q4	397 (14.2)	1.41 (1.20, 1.66)	<0.001	1.35 (1.14, 1.59)	<0.001	1.29 (1.09, 1.53)	0.003	1.28 (1.09, 1.52)	0.004
mRS score 3–6									
Per SD increase	1,514 (13.3)	1.17 (1.11, 1.23)	<0.001	1.14 (1.09, 1.2)	<0.001	1.14 (1.09, 1.20)	<0.001	1.14 (1.08, 1.20)	<0.001
Q1	320 (11.5)	Reference		Reference		Reference		Reference	
Q2	350 (12.2)	1.08 (0.92, 1.26)	0.372	1.09 (0.92, 1.28)	0.316	1.06 (0.90, 1.26)	0.469	1.06 (0.90, 1.26)	0.482
Q3	390 (13.5)	1.20 (1.03, 1.41)	0.021	1.19 (1.02, 1.40)	0.032	1.16 (0.99, 1.37)	0.067	1.18 (1.00, 1.39)	0.052
Q4	454 (15.9)	1.46 (1.25, 1.71)	<0.001	1.40 (1.20, 1.64)	<0.001	1.34 (1.14, 1.58)	<0.001	1.33 (1.13, 1.57)	0.001
*At 1 year*									
Death									
Per SD increase	355 (3.1)	1.13 (1.05, 1.21)	0.001	1.11 (1.04, 1.20)	0.003	1.11 (1.03, 1.19)	0.005	1.11 (1.03, 1.20)	0.010
Q1	73 (2.6)	Reference		Reference		Reference		Reference	
Q2	84 (2.9)	1.12 (0.82, 1.54)	0.469	1.18 (0.86, 1.62)	0.297	1.12 (0.82, 1.54)	0.473	1.13 (0.82, 1.55)	0.448
Q3	89 (3.1)	1.18 (0.87, 1.61)	0.296	1.21 (0.89, 1.65)	0.230	1.15 (0.85, 1.57)	0.367	1.17 (0.86, 1.60)	0.312
Q4	109 (3.8)	1.47 (1.10, 1.98)	0.010	1.50 (1.11, 2.03)	0.008	1.39 (1.03, 1.88)	0.033	1.33 (0.98, 1.80)	0.069
mRS score 3–5									
Per SD increase	1,108 (10.0)	1.13 (1.07, 1.19)	<0.001	1.11 (1.05, 1.17)	<0.001	1.11 (1.05, 1.17)	0.001	1.11 (1.05, 1.17)	0.001
Q1	261 (9.6)	Reference		Reference		Reference		Reference	
Q2	239 (8.6)	0.89 (0.74, 1.07)	0.200	0.90 (0.75, 1.08)	0.265	0.88 (0.73, 1.06)	0.188	0.88 (0.73, 1.07)	0.200
Q3	282 (10.1)	1.05 (0.88, 1.26)	0.565	1.05 (0.88, 1.26)	0.580	1.03 (0.86, 1.23)	0.767	1.04 (0.87, 1.25)	0.680
Q4	326 (11.9)	1.27 (1.07, 1.51)	0.006	1.24 (1.04, 1.48)	0.018	1.19 (0.99, 1.42)	0.057	1.19 (0.99, 1.42)	0.061
mRS score 3–6									
Per SD increase	1,463 (12.8)	1.14 (1.08, 1.20)	<0.001	1.12 (1.07, 1.18)	<0.001	1.12 (1.06, 1.18)	<0.001	1.11 (1.06, 1.17)	<0.001
Q1	334 (12.0)	Reference		Reference		Reference		Reference	
Q2	323 (11.3)	0.94 (0.80, 1.10)	0.427	0.95 (0.81, 1.13)	0.580	0.93 (0.78, 1.10)	0.371	0.92 (0.78, 1.09)	0.340
Q3	371 (12.8)	1.08 (0.92, 1.27)	0.322	1.08 (0.92, 1.27)	0.325	1.06 (0.90, 1.24)	0.518	1.06 (0.90, 1.26)	0.458
Q4	435 (15.3)	1.33 (1.14, 1.54)	<0.001	1.30 (1.11, 1.53)	0.001	1.24 (1.06, 1.46)	0.008	1.24 (1.05, 1.46)	0.011

Hazard ratios were used for death; Odds ratios were used for an mRS score of 3–5 and an mRS score of 3–6. Model 1 was adjusted for age and sex. Model 2 was further adjusted for body mass index, current smoking, heavy drinking, pre-stroke mRS score, TOAST classification, and hypertension, diabetes mellitus, dyslipidemia, previous stroke, and coronary heart disease. Model 3 was further adjusted for antiplatelet agents, anticoagulant agents, estimated glomerular filtration rate, and high-sensitivity C-reactive protein. mRS, modified Rankin Scale. SD, standard deviation; TOAST, Trial of ORG 10172 in Acute Stroke Treatment.

### 3.3 Functional outcome

In total, 1,354 patients (12.0%) had an mRS score of 3–5, and 1,514 patients (13.3%) had an mRS score of 3–6 at 3 months. At the 1-year assessment, 1,108 patients (10.0%) had an mRS score of 3–5, and 1,463 patients (12.8%) had an mRS score of 3–6. The higher the level of ALP grouping, the higher the proportion of patients with high mRS scores (Supplementary Figure S2).

At the 3-month follow-up, compared with the lowest quartile, patients in the highest quartile had a 28 and 33% greater risk of disability and poor functional outcomes, respectively (adjusted OR: 1.28; 95% CI: 1.09–1.52; *p* = 0.004 and 1.33; 95% CI: 1.13–1.57; *p* = 0.001). Similarly, at the 1-year follow-up, the risk of poor functional outcomes was found to increase in the highest quartile compared with the lowest quartile, with adjusted OR 1.24 (95% CI: 1.05–1.46; *p* = 0.011) (Table 2).

At the 3-month follow-up, each SD increase of ALP levels was associated with 12 and 14% higher risk of disability (adjusted OR: 1.12; 95% CI: 1.06–1.18; *p* < 0.001) and poor functional outcomes (adjusted OR: 1.14; 95% CI: 1.08–1.20; *p* < 0.001) in the fully adjusted model, respectively. Similar results were found at the 1-year follow-up (Table 2). In addition, the restricted cubic spline regression analysis showed a linear and positive correlation between serum ALP and functional poor outcomes at 3 months and 1 year (Figures 2B,C,E,F).

### 3.4 TOAST classification

As for TOAST etiologies, for each 1 SD increase of ALP in the “others” subtype, the risk of death at 3 months increased by 19% (adjusted HR: 1.19; 95% CI: 1.04–1.36; *p* = 0.014). Similarly, the risk of death at 1 year increased by 16% (adjusted HR: 1.16; 95% CI: 1.04–1.30; *p* = 0.008) (Supplementary Table S2). However, no correlation with mortality was found among LAA, SVO, and CE subtypes.

**Figure 3:**
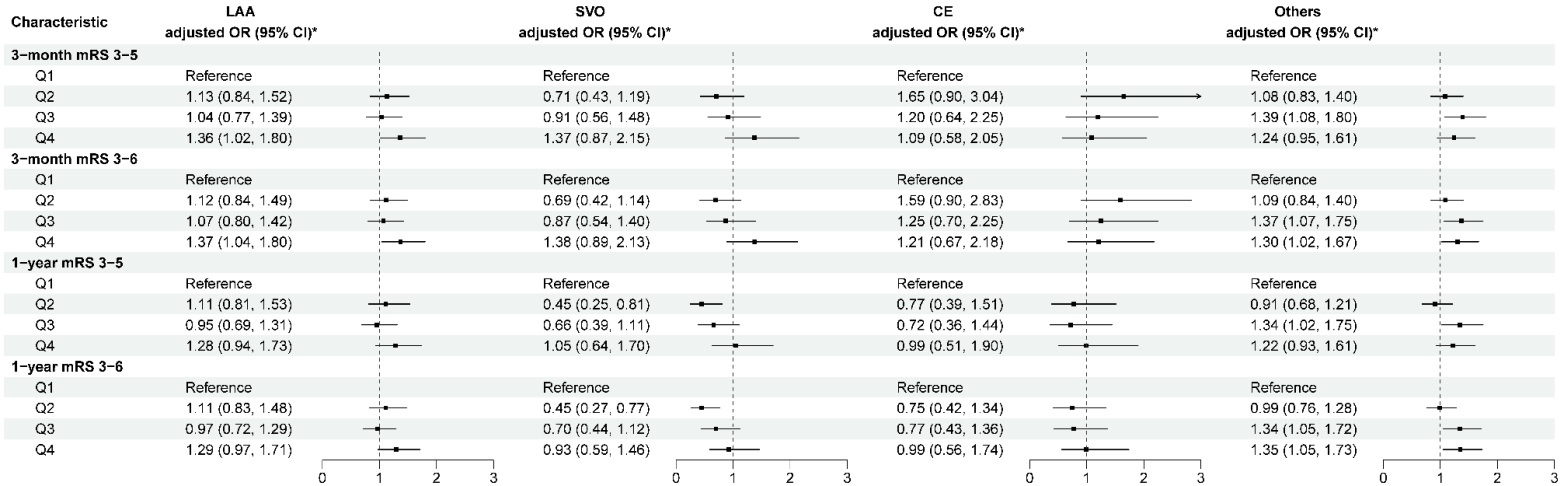
Multivariable analysis of disability and poor functional outcomes according to TOAST classification. *Model 3 is adjusted for age, sex, body mass index, current smoking, heavy drinking, pre-stroke mRS score, TOAST classification, hypertension, diabetes, dyslipidemia, coronary heart disease, previous stroke, antiplatelet agents, anticoagulant agents, estimated glomerular filtration rate, and high-sensitivity C-reactive protein. CE, cardioembolism; LAA, large-artery atherosclerosis; mRS, modified Rankin Scale; others, other determined etiology and undetermined etiology; SVO, small-vessel occlusion; TOAST, Trial of ORG 10172 in Acute Stroke Treatment.

After adjusting for potential confounding factors (Model 3), in the “others” subtype, higher levels of ALP were associated with poor functional outcomes at 3 months (OR per SD: 1.15 [95% CI: 1.06–1.24; *p* = 0.001]) and 1 year (OR per SD: 1.16; 95% CI: 1.07–1.26, *p* < 0.001). Similar results were found for disability. In LAA and SVO subtypes, elevated ALP levels were associated with an increased risk of poor functional outcomes at 3 months, with ORs were 1.11 (95% CI: 1.01–1.22, *p* = 0.023) and 1.18 (95% CI: 1.04–1.34, *p* = 0.010), respectively. Similar trends were observed for disability. Furthermore, compared to the lowest quartile, higher levels of ALP were associated with an increased risk of disability and poor functional outcomes in patients with LAA at 3 months (*p* < 0.05), and there was also an increased risk of poor functional outcomes in patients with the “others” subtype at 3 months and 1 year (*p* < 0.05) (Figure 3; Supplementary Table S2). However, no correlation between disability and poor functional prognosis was found in the SVO and CE subtypes.

## 4 Discussion

This study revealed a positive association between elevated ALP levels and an increased risk of mortality, disability, and poor functional outcomes in patients with AIS. Specifically, elevated ALP levels were linked to adverse clinical outcomes in the others subtype and were correlated with an increased risk of disability and poor functional outcomes in the LAA and SVO subtypes at 3 months. These findings offer new insights into the role of ALP levels in the AIS prediction. The Chinese Stroke Registry II and the Xi’an multicenter study reported that higher ALP levels were associated with increased patient mortality, without identifying a linear correlation (17, 18). Conversely, a Korean single-center study indicated a positive linear correlation between elevated ALP levels and mortality (16). In line with these findings, our study indicated that elevated ALP levels were associated with an increased risk of all-cause mortality, with a linear correlation observed. The differences in the studies primarily resulted from the included populations. The linear correlation study focused on IS patients, while the non-linear correlation studies included mixed stroke (including ischemic stroke and hemorrhagic stroke) patients. Ryu et al. also revealed that hemorrhagic stroke patients in the elevated ALP group had a higher risk of death than ischemic patients, which may affect the linear and non-linear relationships (16). Furthermore, Zhong et al. (22) observed similar results in their study of 2,944 enrolled AIS patients. We also performed an analysis of poor functional outcomes, and similar to Zhu et al. and Kim et al., we found that higher ALP levels were associated with an increased risk of poor functional outcomes (11, 19). However, Guo et al. and Liu et al. found that elevated ALP levels were not associated with poor functional outcomes in stroke patients (18, 20). The difference in conclusions may be related to the participant characteristics and sample size. Furthermore, we analyzed the disability and discovered that elevated levels of ALP could serve as a predictor for disability in patients with AIS and TIA.

In CVD studies, the elevated mortality associated with increased ALP levels was related to atherosclerosis (23). Unlike CVD, IS is a heterogeneous disease with a distinct pathogenesis. The mechanism between elevated serum ALP and prognosis in patients with IS remains unclear; no studies have investigated the role of ALP in different subtypes of IS. However, in this study, an increase in ALP was not found to be associated with all-cause mortality in patients with the LAA subtype, and only LAA and SVO subtypes were associated with poor functional outcomes and disability during the short-term follow-up. Kim et al. also found that higher ALP levels were not associated with cerebral atherosclerosis (19). Therefore, the association between higher ALP levels and an increased risk of adverse outcomes in stroke patients might be unrelated to the mechanism of atherosclerosis. This study discovered a significant association between elevated levels of ALP and the prognosis of patients with an undetermined etiology stroke subtype. Furthermore, it revealed that patients with a history of coronary heart disease exhibited elevated serum ALP levels. The occurrence of adverse clinical outcomes may be related to unstable and easy detachment of calcified plaques or cardioembolism (24, 25), which requires further research.

Systemic inflammation has been recognized as a significant factor influencing the short-term and long-term outcomes of patients with stroke (26, 27). The pathophysiological mechanisms underlying the adverse clinical outcomes in patients with elevated ALP levels may be associated with the interplay between elevated ALP, neuroinflammation, blood–brain barrier (BBB) permeability, and vascular homeostasis (28). The immune rescue mechanism of neuroinflammation was reported to be activated after cerebral ischemia (29), resulting in an increase in ALP (7, 30, 31), which was consistent with the current proposal that peripheral immunity is involved in complex brain immune networks (32). Furthermore, tissue-non-specific alkaline phosphatase (TNAP), the isoenzyme of ALP, is abundant in brain endothelial cells and neurons (33) and regulates neuroinflammatory responses (34, 35). After the breakdown of the BBB, TNAP is lost to the periphery, and the decrease in TNAP levels further exacerbates brain damage (36, 37). Thus, we hypothesized that elevated serum ALP levels in the acute phase could indicate a significant depletion of ALP in the brain, reflecting the extent of neurological impairment and ultimately leading to a poor prognosis. The potential of oral ALP or TNAP administration for the treatment of nerve damage following IS presents a compelling area for future investigation.

This study is a large-scale, multicenter prospective study with a substantial sample size, including patients from 201 hospitals, which enhances the generalizability of the research findings. This study explores, for the first time, the impact of ALP on various TOAST subtypes. However, the study also has some limitations. First, this was an observational study, controlling for some important potential confounding factors in the multivariable adjustment model; however, it is still difficult to entirely eliminate the possibility of residual confounding. Second, we did not collect information on vitamin D deficiency in our study, despite the known impact of vitamin D on serum ALP levels. To minimize the potential confounding effects, we collected blood samples at a predetermined time (the next morning after the admission with overnight fasting). Third, our study only examined ALP levels in the acute phase and did not assess their continuity over time. Therefore, it remains unclear whether changes in ALP levels may in turn impact the outcomes of IS. Fourth, the types of ALP isoenzymes have not been evaluated, and it was not possible to assess which types of ALP are associated with adverse stroke outcomes. Further studies are needed to confirm the role of isoenzymes in AIS and TIA, which might provide more valuable information for understanding the mechanism of ALP on clinical outcomes. Additionally, since all participants in the study were Chinese, the generalizability to other races and ethnicities may be limited.

## 5 Conclusion

In summary, this study showed that elevated ALP levels were associated with an increased risk of all-cause mortality, disability, and poor function outcomes in patients with IS. Furthermore, heterogeneity was observed among the subtypes of different stroke etiologies.

## Data availability statement

The original contributions presented in the study are included in the article/Supplementary material, further inquiries can be directed to the corresponding authors.

## Ethics statement

The studies involving humans were approved by the Ethics Committee of Beijing Tiantan Hospital (No. KY2015-001-01). The studies were conducted in accordance with the local legislation and institutional requirements. The participants provided their written informed consent to participate in this study.

## Author contributions

ZW: Conceptualization, Investigation, Writing – original draft, Writing – review & editing. JLi: Methodology, Writing – original draft, Writing – review & editing. JJ: Data curation, Project administration, Supervision, Writing – review & editing. ZZ: Data curation, Project administration, Supervision, Writing – review & editing. QX: Data curation, Formal analysis, Writing – review & editing. TL: Data curation, Project administration, Supervision, Writing – review & editing. JLin: Data curation, Formal analysis, Investigation, Writing – review & editing. YJ: Data curation, Methodology, Writing – review & editing. YW: Conceptualization, Supervision, Writing – review & editing. AW: Conceptualization, Methodology, Supervision, Writing – review & editing. XM: Conceptualization, Methodology, Project administration, Supervision, Writing – review & editing.
