# Supplementary material for: Impact of alkaline phosphatase on clinical outcomes in patients with ischemic stroke: a nationwide registry analysis
Source: Front Neurol. 2024 Feb 14;15:1336069. doi: 10.3389/fneur.2024.1336069 (PMC10899335; doi:10.3389/fneur.2024.1336069)
Supplement: Supplementary file 1 [file Data_Sheet_1.pdf]

SUPPLEMENTAL MATERIAL

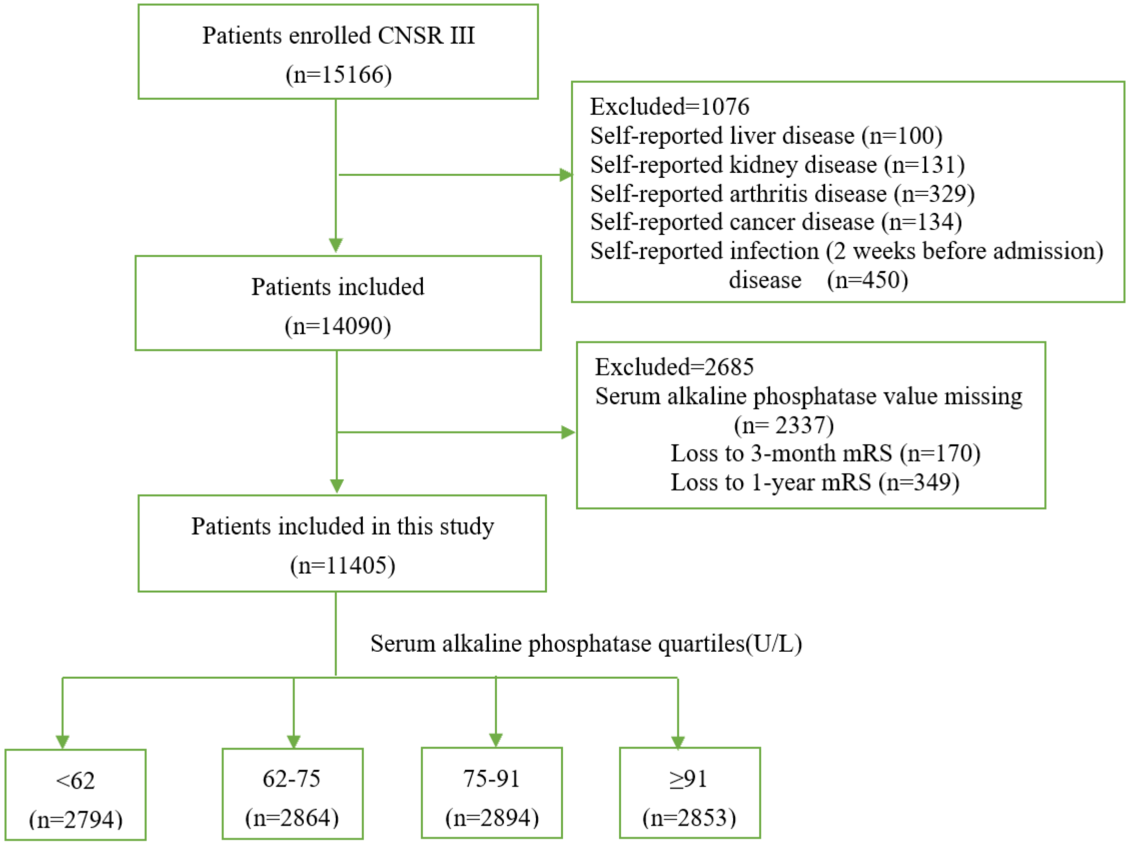

Figure S1. Patients flow diagram. CNSR-III, China National Stroke Registry III.

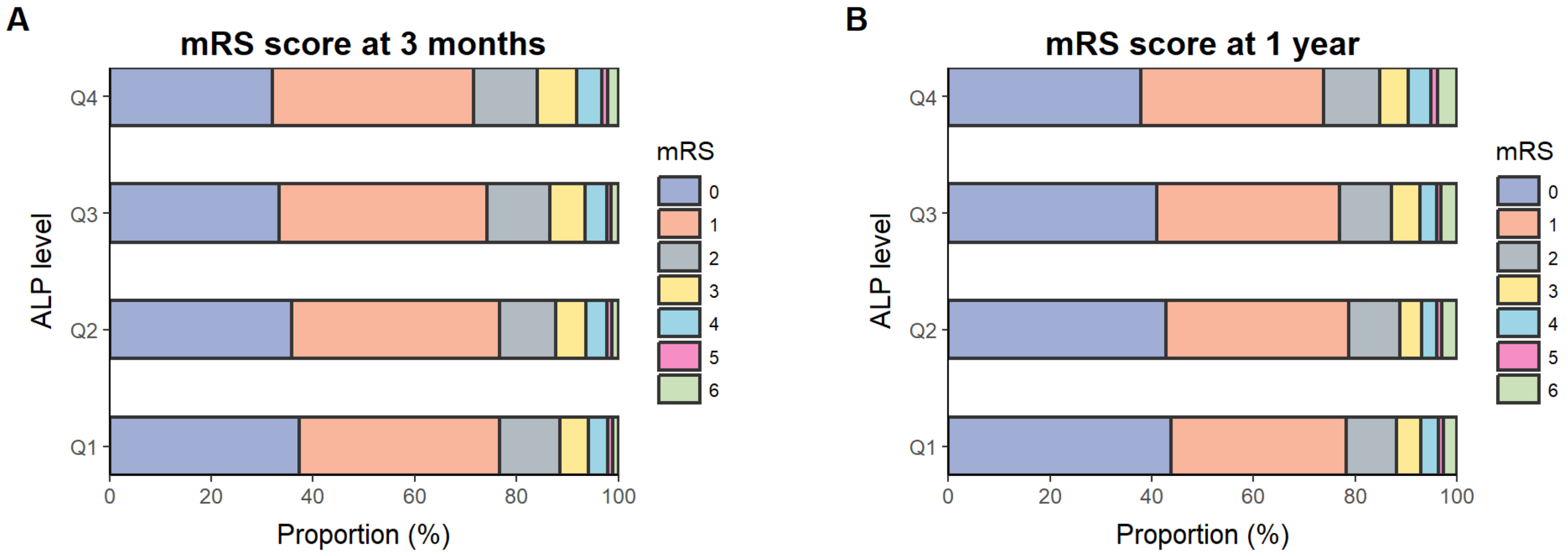

Figure S2. Proportion of mRS score by quartile of ALP levels. Distribution of mRS scores at 3 months (A) and 1 year (B). The mRS score ranges from 0 to 6 (0, no symptoms; 1, no clinical disability; 2, slight disability; 3, moderate disability; 4, moderately severe disability; 5, severe disability; 6, death). Abbreviations: ALP, alkaline phosphatase; mRS, modified Rankin Scale.

Table S1. Comparison between participants who were included and those who were excluded

	Overall (n=15166)	Excluded (n=3761)	Included (n=11405)	P
AIS	13982 (92.2)	3457 (91.9)	10525 (92.3)	0.489
Age, years	63.0 [54.0, 70.0]	62.0 [54.0, 70.0]	63.0 [54.0, 70.0]	0.657
Men, n (%)	10364 (68.3)	2580 (68.6)	7784 (68.3)	0.706
Body mass index, kg/m^2	24.5 [22.6, 26.6]	24.6 [22.7, 26.7]	24.5 [22.6, 26.5]	0.050
Current smoking, n (%)	4752 (31.3)	1147 (30.5)	3605 (31.6)	0.210
Heavy drinking, n (%)	2126 (14.0)	498 (13.2)	1628 (14.3)	0.120
Pre-stroke mRS score	0.0 [0.0, 1.0]	0.0 [0.0, 1.0]	0.0 [0.0, 1.0]	0.391
NIHSS score at admission	3.0 [1.0, 6.0]	3.0 [1.0, 6.0]	3.0 [1.0, 6.0]	0.001
TOAST classification, n (%)				0.457
Large-artery atherosclerosis	3856 (25.4)	958 (25.5)	2898 (25.4)	
Cardioembolism	917 (6.0)	226 (6.0)	691 (6.1)	
Small-vessel occlusion	3165 (20.9)	773 (20.6)	2392 (21.0)	
Other determined etiology	182 (1.2)	35 (0.9)	147 (1.3)	
Undetermined etiology	7046 (46.5)	1769 (47.0)	5277 (46.3)	
Medical history, n (%)				
Hypertension	9494 (62.6)	2371 (63.0)	7123 (62.5)	0.532
Diabetes mellitus	3510 (23.1)	848 (22.5)	2662 (23.3)	0.328
Dyslipidemia	1191 (7.9)	330 (8.8)	861 (7.5)	0.017
Previous ischemic stroke	3355 (22.1)	828 (22.0)	2527 (22.2)	0.874
Coronary heart disease	1608 (10.6)	419 (11.1)	1189 (10.4)	0.228
Treatment in hospital, n (%)				
Intravenous Thrombolysis	1303 (8.6)	354 (9.4)	949 (8.3)	0.042
Mechanical thrombectomy	39 (0.3)	7 (0.2)	32 (0.3)	0.420
Antiplatelet agents	14613 (96.4)	3611 (96.0)	11002 (96.5)	0.197
Anticoagulant agents	1546 (10.2)	355 (9.4)	1191 (10.4)	0.082
Lipid-lowering agents	14506 (95.7)	3617 (96.2)	10889 (95.5)	0.084
Laboratory tests				
TC, mmol/L	4.1 [3.4, 4.9]	4.1 [3.4, 4.9]	4.2 [3.4, 4.9]	0.379
HDL-C, mmol/L	1.1 [0.9, 1.3]	1.1 [0.9, 1.3]	1.1 [0.9, 1.3]	<0.001
LDL-C, mmol/L	2.4 [1.8, 3.1]	2.4 [1.8, 3.1]	2.5 [1.9, 3.1]	0.025
TG, mmol/L	1.4 [1.0, 1.9]	1.4 [1.0, 1.9]	1.4 [1.0, 1.9]	0.270
ALT, U/L	18.0 [13.0, 26.0]	18.0 [13.0, 27.0]	18.0 [13.0, 25.0]	0.002
AST, U/L	19.0 [16.0, 24.0]	19.8 [16.0, 24.7]	19.0 [16.0, 24.0]	<0.001
eGFR	93.2 [81.6, 102.0]	93.3 [80.2, 102.6]	93.2 [82.0, 101.8]	0.590
hs-CRP	1.8 [0.8, 4.8]	2.2 [0.9, 5.7]	1.8 [0.8, 4.6]	<0.001

Abbreviations: AIS, acute ischemic stroke; ALT, alanine aminotransferase; AST, aspartate aminotransferase; eGFR, estimated glomerular filtration rate; HDL-C, high-density lipoprotein cholesterol; hs-CRP, high sensitivity C-reactive protein; LDL, low-density lipoprotein cholesterol; mRS, modified Rankin Scale; NIHSS, National Institutes of Health Stroke Scale; TC, total cholesterol; TG, triglycerides; TOAST, Trial of ORG 10172 in Acute Stroke Treatment.

Table S2. Associations of alkaline phosphatase with all-cause mortality, disability, and poor functional outcomes according to TOAST classification in model 3

	LAA	*P*	SVO	*P*	CE	*P*	Others	*P*
**At 3 months**								
Death								
Per SD increase	1.08 (0.86, 1.36)	0.487	1.12 (0.59, 2.11)	0.736	1.10 (0.87, 1.40)	0.426	1.19 (1.04, 1.36)	0.014
Q1	Reference		Reference		Reference		Reference	
Q2	0.97 (0.41, 2.30)	0.945	0.29 (0.02, 3.48)	0.330	1.36 (0.35, 5.21)	0.655	1.23 (0.60, 2.51)	0.577
Q3	1.37 (0.61, 3.05)	0.443	0.25 (0.02, 3.06)	0.276	1.70 (0.46, 6.18)	0.424	1.10 (0.53, 2.27)	0.795
Q4	1.28 (0.57, 2.84)	0.551	0.88 (0.14, 5.55)	0.892	1.95 (0.55, 6.96)	0.304	1.74 (0.90, 3.36)	0.102
mRS score 3-5								
Per SD increase	1.11 (1.01, 1.22)	0.031	1.18 (1.04, 1.34)	0.012	1.09 (0.94, 1.26)	0.234	1.13 (1.03, 1.23)	0.007
Q1	Reference		Reference		Reference		Reference	
Q2	1.13 (0.84, 1.52)	0.419	0.71 (0.43, 1.19)	0.195	1.65 (0.90, 3.04)	0.108	1.08 (0.83, 1.40)	0.579
Q3	1.04 (0.77, 1.39)	0.815	0.91 (0.56, 1.48)	0.709	1.20 (0.64, 2.25)	0.569	1.39 (1.08, 1.80)	0.010
Q4	1.36 (1.02, 1.80)	0.035	1.37 (0.87, 2.15)	0.172	1.09 (0.58, 2.05)	0.789	1.24 (0.95, 1.61)	0.108
mRS score 3-6								
Per SD increase	1.11 (1.01, 1.22)	0.023	1.18 (1.04, 1.34)	0.010	1.11 (0.96, 1.28)	0.171	1.15 (1.06, 1.24)	0.001
Q1	Reference		Reference		Reference		Reference	
Q2	1.12 (0.84, 1.49)	0.447	0.69 (0.42, 1.14)	0.148	1.59 (0.90, 2.83)	0.112	1.09 (0.84, 1.40)	0.519
Q3	1.07 (0.80, 1.42)	0.653	0.87 (0.54, 1.40)	0.570	1.25 (0.70, 2.25)	0.453	1.37 (1.07, 1.75)	0.011
Q4	1.37 (1.04, 1.80)	0.025	1.38 (0.89, 2.13)	0.153	1.21 (0.67, 2.18)	0.526	1.30 (1.02, 1.67)	0.036
**At 1 year**								
Death								
Per SD increase	1.05 (0.88, 1.24)	0.597	0.84 (0.54, 1.31)	0.439	1.04 (0.82, 1.32)	0.738	1.16 (1.04, 1.30)	0.008
Q1	Reference		Reference		Reference		Reference	
Q2	1.11 (0.64, 1.94)	0.707	0.50 (0.16, 1.55)	0.232	0.93 (0.40, 2.16)	0.864	1.31 (0.81, 2.10)	0.273
Q3	1.06 (0.60, 1.85)	0.851	0.96 (0.36, 2.53)	0.932	1.15 (0.51, 2.58)	0.734	1.34 (0.83, 2.16)	0.229
Q4	1.24 (0.73, 2.13)	0.429	0.47 (0.15, 1.48)	0.195	1.06 (0.47, 2.40)	0.887	1.63 (1.04, 2.58)	0.035
mRS score 3-5								
Per SD increase	1.05 (0.95, 1.17)	0.324	1.13 (0.97, 1.30)	0.117	1.16 (0.99, 1.36)	0.065	1.13 (1.03, 1.24)	0.008
Q1	Reference		Reference		Reference		Reference	
Q2	1.11 (0.81, 1.53)	0.512	0.45 (0.25, 0.81)	0.008	0.77 (0.39, 1.51)	0.445	0.91 (0.68, 1.21)	0.512
Q3	0.95 (0.69, 1.31)	0.747	0.66 (0.39, 1.11)	0.118	0.72 (0.36, 1.44)	0.352	1.34 (1.02, 1.75)	0.034
Q4	1.28 (0.94, 1.73)	0.119	1.05 (0.64, 1.70)	0.858	0.99 (0.51, 1.90)	0.965	1.22 (0.93, 1.61)	0.154

mRS score 3-6								
Per SD increase	1.05 (0.96, 1.16)	0.284	1.09 (0.94, 1.26)	0.254	1.14 (0.98, 1.32)	0.085	1.16 (1.07, 1.26)	<0.001
Q1	Reference		Reference		Reference		Reference	
Q2	1.11 (0.83, 1.48)	0.498	0.45 (0.27, 0.77)	0.003	0.75 (0.42, 1.34)	0.335	0.99 (0.76, 1.28)	0.916
Q3	0.97 (0.72, 1.29)	0.817	0.70 (0.44, 1.12)	0.135	0.77 (0.43, 1.36)	0.361	1.34 (1.05, 1.72)	0.018
Q4	1.29 (0.97, 1.71)	0.077	0.93 (0.59, 1.46)	0.751	0.99 (0.56, 1.74)	0.973	1.35 (1.05, 1.73)	0.018

Note: Hazard ratios were used for death; Odds ratios were used for an mRS score of 3–5 and an mRS score of 3–6. Model 3 was adjusted for age, sex, body mass index, current smoking, alcohol consumption, mRS score on admission, TOAST classification, hypertension, diabetes, dyslipidemia, coronary heart disease, and previous stroke, antiplatelet agents, anticoagulant agents, estimated glomerular filtration rate, and high-sensitivity C-reactive protein. Abbreviations: CE, cardioembolism; mRS, modified Rankin Scale; LAA, large-artery atherosclerosis; Others, other determined etiology and undetermined etiology; SVO, small-vessel occlusion; TOAST, Trial of ORG 10172 in Acute Stroke Treatment.

Table S3. Associations of alkaline phosphatase with all-cause mortality, disability, and poor functional outcomes after exclude TIA patients

	**Events, n (%)**	**Unadjusted**	***P***	**Model 1**	***P***	**Model 2**	***P***	**Model 3**	***P***
**At 3 months**									
Death									
Per SD increase	156 (1.5)	1.18 (1.09, 1.27)	<0.001	1.15 (1.06, 1.25)	0.001	1.15 (1.05, 1.25)	0.001	1.15 (1.05, 1.26)	0.002
Q1	30 (1.2)	Reference		Reference		Reference		Reference	
Q2	33 (1.3)	1.05 (0.64, 1.73)	0.832	1.12 (0.68, 1.84)	0.657	1.07 (0.65, 1.76)	0.778	1.07 (0.65, 1.76)	0.786
Q3	39 (1.5)	1.22 (0.76, 1.97)	0.406	1.24 (0.77, 1.99)	0.381	1.21 (0.75, 1.95)	0.441	1.21 (0.75, 1.96)	0.426
Q4	54 (2.0)	1.71 (1.10, 2.68)	0.018	1.70 (1.08, 2.67)	0.022	1.61 (1.02, 2.54)	0.039	1.52 (0.96, 2.40)	0.077
mRS score 3-5									
Per SD increase	1332 (12.9)	1.15 (1.09, 1.21)	<0.001	1.12 (1.06, 1.18)	<0.001	1.12 (1.06, 1.18)	<0.001	1.12 (1.06, 1.18)	<0.001
Q1	285 (11.4)	Reference		Reference		Reference		Reference	
Q2	311 (11.9)	1.05 (0.89, 1.25)	0.543	1.07 (0.90, 1.27)	0.451	1.06 (0.89, 1.26)	0.537	1.06 (0.88, 1.26)	0.550

Q3	344 (13.0)	1.16 (0.98, 1.37)	0.081	1.15 (0.97, 1.36)	0.102	1.13 (0.95, 1.34)	0.156	1.15 (0.96, 1.36)	0.124
Q4	392 (15.0)	1.37 (1.16, 1.61)	<0.001	1.31 (1.11, 1.55)	0.002	1.26 (1.06, 1.50)	0.007	1.25 (1.05, 1.48)	0.010
mRS score 3-6									
Per SD increase	1488 (14.1)	1.16 (1.10, 1.22)	<0.001	1.13 (1.08, 1.19)	<0.001	1.13 (1.08, 1.19)	<0.001	1.13 (1.07, 1.19)	<0.001
Q1	315 (12.5)	Reference		Reference		Reference		Reference	
Q2	344 (13.1)	1.06 (0.90, 1.24)	0.519	1.07 (0.91, 1.27)	0.408	1.06 (0.89, 1.25)	0.526	1.05 (0.89, 1.25)	0.567
Q3	383 (14.3)	1.17 (1.00, 1.37)	0.055	1.16 (0.98, 1.36)	0.076	1.14 (0.96, 1.34)	0.127	1.15 (0.97, 1.36)	0.101
Q4	446 (16.7)	1.41 (1.21, 1.65)	<0.001	1.35 (1.15, 1.59)	<0.001	1.30 (1.11, 1.53)	0.002	1.29 (1.09, 1.52)	0.003
**At 1 year**									
Death									
Per SD increase	342 (3.3)	1.12 (1.04, 1.21)	0.002	1.11 (1.03, 1.19)	0.006	1.10 (1.02, 1.19)	0.011	1.10 (1.02, 1.19)	0.018
Q1	72 (2.8)	Reference		Reference		Reference		Reference	
Q2	78 (3.0)	1.04 (0.75, 1.43)	0.814	1.11 (0.80, 1.53)	0.530	1.06 (0.77, 1.46)	0.737	1.06 (0.77, 1.47)	0.705
Q3	88 (3.3)	1.15 (0.84, 1.57)	0.370	1.19 (0.87, 1.62)	0.279	1.14 (0.83, 1.56)	0.416	1.16 (0.85, 1.59)	0.355
Q4	104 (3.9)	1.38 (1.02, 1.86)	0.036	1.42 (1.05, 1.93)	0.024	1.32 (0.97, 1.79)	0.076	1.26 (0.92, 1.71)	0.145
mRS score 3-5									
Per SD increase	1083 (10.6)	1.12 (1.06, 1.18)	<0.001	1.10 (1.04, 1.16)	0.001	1.10 (1.04, 1.17)	0.001	1.10 (1.04, 1.17)	0.001
Q1	253 (10.3)	Reference		Reference		Reference		Reference	
Q2	234 (9.1)	0.88 (0.73, 1.06)	0.171	0.90 (0.74, 1.08)	0.257	0.88 (0.73, 1.07)	0.210	0.89 (0.73, 1.07)	0.215
Q3	275 (10.6)	1.03 (0.86, 1.24)	0.734	1.03 (0.86, 1.24)	0.730	1.01 (0.84, 1.22)	0.886	1.03 (0.85, 1.24)	0.792
Q4	321 (12.5)	1.25 (1.05, 1.49)	0.013	1.22 (1.02, 1.46)	0.030	1.18 (0.98, 1.41)	0.082	1.17 (0.98, 1.41)	0.087
mRS score 3-6									
Per SD increase	1425 (13.5)	1.13 (1.08, 1.19)	<0.001	1.11 (1.06, 1.17)	<0.001	1.11 (1.06, 1.17)	<0.001	1.11 (1.05, 1.17)	<0.001
Q1	325 (12.8)	Reference		Reference		Reference		Reference	
Q2	312 (11.8)	0.91 (0.77, 1.08)	0.270	0.94 (0.79, 1.11)	0.439	0.91 (0.77, 1.08)	0.294	0.90 (0.76, 1.08)	0.255
Q3	363 (13.5)	1.06 (0.90, 1.24)	0.476	1.06 (0.90, 1.25)	0.459	1.04 (0.88, 1.23)	0.644	1.05 (0.89, 1.24)	0.577
Q4	425 (15.9)	1.29 (1.10, 1.50)	0.002	1.27 (1.08, 1.49)	0.004	1.21 (1.03, 1.43)	0.021	1.21 (1.02, 1.42)	0.028

Note: Hazard ratios were used for death; Odds ratios were used for an mRS score of 3–5 and an mRS score of 3–6. Model 1 was adjusted for age and sex. Model 2 was further adjusted for body mass index, current smoking, heavy drinking, pre-stroke mRS score, TOAST classification, and hypertension, diabetes mellitus, dyslipidemia, previous stroke, and coronary heart disease. Model 3 was further adjusted for antiplatelet agents, anticoagulant agents, estimated glomerular filtration rate, and high sensitivity C-reactive protein. Abbreviations: mRS, modified Rankin Scale; SD, standard deviation; TIA, transient ischemic attack (TIA); TOAST, Trial of ORG 10172 in Acute Stroke Treatment.
